# Anti-Inflammatory Effects of β-Cryptoxanthin on 5-Fluorouracil-Induced Cytokine Expression in Human Oral Mucosal Keratinocytes

**DOI:** 10.3390/molecules28072935

**Published:** 2023-03-24

**Authors:** Hironaka Yamanobe, Kenta Yamamoto, Saki Kishimoto, Kei Nakai, Fumishige Oseko, Toshiro Yamamoto, Osam Mazda, Narisato Kanamura

**Affiliations:** 1 Department of Dental Medicine, Kyoto Prefectural University of Medicine, Kyoto 602-8566, Japan; 2 Department of Immunology, Kyoto Prefectural University of Medicine, Kyoto 602-8566, Japan

**Keywords:** β-cryptoxanthin, 5-fluorouracil, oral mucosal keratinocytes

## Abstract

Oral mucositis is a typical adverse effect of chemotherapy, causing oral pain that significantly reduces the patient’s quality of life. β-cryptoxanthin (β-cry) is a carotenoid abundant in citrus fruits with antioxidant and anti-inflammatory effects. However, the β-cry effect on oral mucositis remains unclear. We investigated the effects of 5-fluorouracil (5-FU) and β-cry on human normal oral mucosal keratinocytes (hOMK). hOMK was seeded on a culture plate and cultured with 5-FU and β-cry. The cell number, mRNA expression of inflammatory cytokines and matrix metalloproteinases (MMPs), and production of inflammatory cytokines in hOMK were evaluated. Additionally, the cell count and inflammatory cytokine production were analyzed when hOMK was co-stimulated with *Porphyromonas gingivalis* lipopolysaccharide (*P. gingivalis* LPS) in addition to 5-FU. The numbers of hOMK significantly reduced with 5-FU stimulation, whereas it increased with β-cry treatment. mRNA expression of interleukin (IL)-6, IL-8, metalloproteinase (MMP)-2, and MMP-9 and protein production of IL-6 and IL-8 in hOMK were augmented on 5-FU stimulation. Simultaneously, β-cry treatment significantly suppressed IL-8 and MMP-9 mRNA expression, and IL-8 production was induced on 5-FU stimulation. Co-stimulation with *P. gingivalis* LPS and 5-FU enhanced IL-6 and IL-8 production in hOMK. β-cry could enhance cell proliferation and suppress 5-FU-induced expression of inflammatory cytokines and MMP in hOMK. Thus, β-cry can alleviate the symptoms of chemotherapy-induced oral mucositis, and its combination with oral care is effective in managing oral mucositis.

## 1. Introduction

The maintenance and management of oral hygiene and function are essential for the prevention of oral mucositis and aspiration pneumonia in patients undergoing cancer chemotherapy, radiation therapy, and surgery under general anesthesia [1]. Oral mucositis causes significant oral pain and dysphagia, which negatively impacts the patient’s quality of life by affecting the patient’s nutritional status and causing difficulty in speaking. In addition, oral mucositis can lead to infection, worsening of the patient’s general condition, and reduction in the dose of chemotherapeutic agents that can affect the length of hospital stay and patient survival [2,3,4]. The use of 5-fluorouracil (5-FU) has led to a reduction in the dose or discontinuation of chemotherapeutic agents in more than 15% of patients due to grade 3 to 4 oral mucositis [5]. Given that cancer continues to be an important cause of death worldwide, conventional anticancer therapies are widely used. Among these anticancer drugs, treatment with 5-FU is the most efficient and broad-spectrum agent, with fluoropyrimidine therapy estimated to be administered to approximately 2 million patients annually; furthermore, 5FU and capecitabine are on the WHO Model List of Essential Medicines [6]. 5FU is also most commonly used for oral squamous cell carcinoma (OSCC) [7,8]. Squamous cell carcinoma of the head and neck is the sixth most common cancer worldwide, killing approximately 350,000 people each year [9,10].

Several factors are involved in the pathogenesis of oral mucositis, and the detailed mechanisms have not yet been fully elucidated. At present, in addition to the direct damage to keratinocytes caused by chemotherapeutic agents such as 5-FU, oral mucositis is thought to be caused by the cytotoxicity of reactive oxygen species (ROS) in the oral mucosal cells, as well as excessive production of inflammatory cytokines and matrix metalloproteinases (MMPs) due to activation of transcriptional regulators, such as NF-kB and MAP kinase [11,12,13]. In addition, chemotherapeutic agents have disrupted the epithelial barrier and weakened the immune cells in some cases, resulting in increased susceptibility to infections, leading to secondary infections by oral bacteria and prolonged oral mucositis.

Various methods have been used to treat oral mucositis, including laser irradiation, mouthwashes with local anesthetics, and cryotherapy [14,15,16,17]; however, it is primarily managed by symptomatic treatment. Thus, more effective prevention and treatment methods are required.

β-cryptoxanthin (β-cry), a carotenoid found in tangerines, persimmons, and bell peppers, is an essential nutrient that supports immune system growth, development, and maintenance as a provitamin A. Prolonged consumption of β-cry-rich foods gradually increases blood β-cry levels in humans and animals [18], and has been reported to inhibit carcinogenesis, support DNA repair, have antioxidant and anti-inflammatory effects [19,20,21], and prevent osteoporosis and osteoarthritis of the knee [22,23]. In addition, the mean plasma-depletion half-life of cryptoxanthin is <12 days [24], and the stability of persimmon carotenoids is 61 to 74%, depending on the digestion phase, with (all-E)-β-cryptoxanthin and (all-E)-antheraxanthin 3-O-palmitate being the most stable carotenoids [25]. Previous studies showed that β-cry has an anti-inflammatory effect on the periodontal ligament cells with respect to *Porphyromonas gingivalis* (*P. gingivalis*)-induced inflammation [26]. However, the effect of β-cry on oral mucositis remains unclear. Therefore, this study investigated the effect of β-cry on oral mucosal keratinocytes using 5-FU, which is frequently used in chemotherapy for squamous cell carcinoma of the head and neck region and causes oral mucositis in a high percentage of patients.

## 2. Results

### 2.1. Cell Growth and Morphology of 5-FU and β-Cry Treated Human Normal Oral Mucosal Keratinocytes (hOMK)

**Figure 1:**
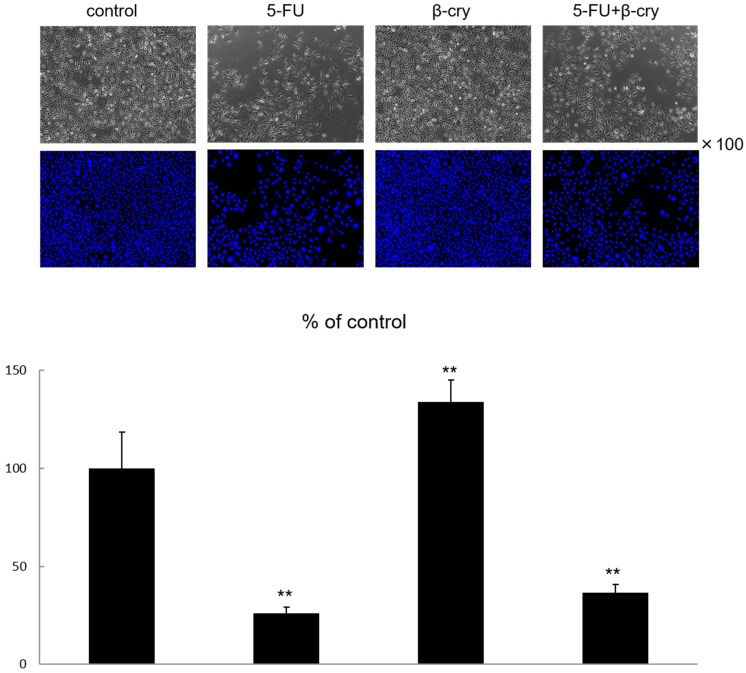
Morphological changes and cell number of hOMK after treatment with 5-FU. The cell number of 5-FU-treated hOMK was significantly lower than that of the control (*p* < 0.01), while the cell number of β-cry-treated hOMK was significantly higher than that of the control (*p* < 0.01). ** *p* < 0.01 vs. control.

hOMK were stimulated with 5-FU at concentrations ranging from 0.01 μg/mL to 10 μg/mL for 48 h, and the subsequent cellular activity of hOMK was examined by WST assay. 5-FU concentration above 1 μg/mL showed a significant decrease in cell growth (48.18% ± 10.62) (Supplementary Materials Figure S1). Therefore, future experiments were conducted using 5-FU at a concentration of 0.5 μg/mL and 7 days after the start of chemotherapy, when oral mucositis was more likely to develop [13]. Morphological changes in hOMK after treatment with 5-FU and β-cry significantly decreased the long–short axis ratio in the 5FU-stimulated and 5FU+β-cry-stimulated groups compared to the control group (Supplementary Materials Figure S3). A report on changes in cell morphology of lung cells induced by cytotoxic damage with an anti-cancer drug, which can interfere with the growth and proliferation of cells and tissues, showed that the cells lost their spindle-shaped morphology compared to control cells, with larger cells, rounder membranes, and other structures [27]. Therefore, similar to our results, the long–short axis ratio was reduced in cells that have undergone cytotoxicity compared to control cells. The hOMK stimulated with 0.5 μg/mL 5-FU for 7 days resulted in significant inhibition of cell proliferation (25.93% ± 3.21) compared with that in the control group (Figure 1). In contrast, the stimulation of hOMK with β-cry for 7 days showed a significant increase in cell proliferation (134.13% ± 10.96) compared with that in the control group (Figure 1). In the 5-FU+β-cry group, cell proliferation was slightly increased (36.66% ± 4.26) but not significantly compared with that in the 5-FU stimulated group.

### 2.2. Effects of β-Cry on 5-FU-Induced mRNA Expression of Inflammatory Cytokines and MMPs in hOMK

**Figure 2:**
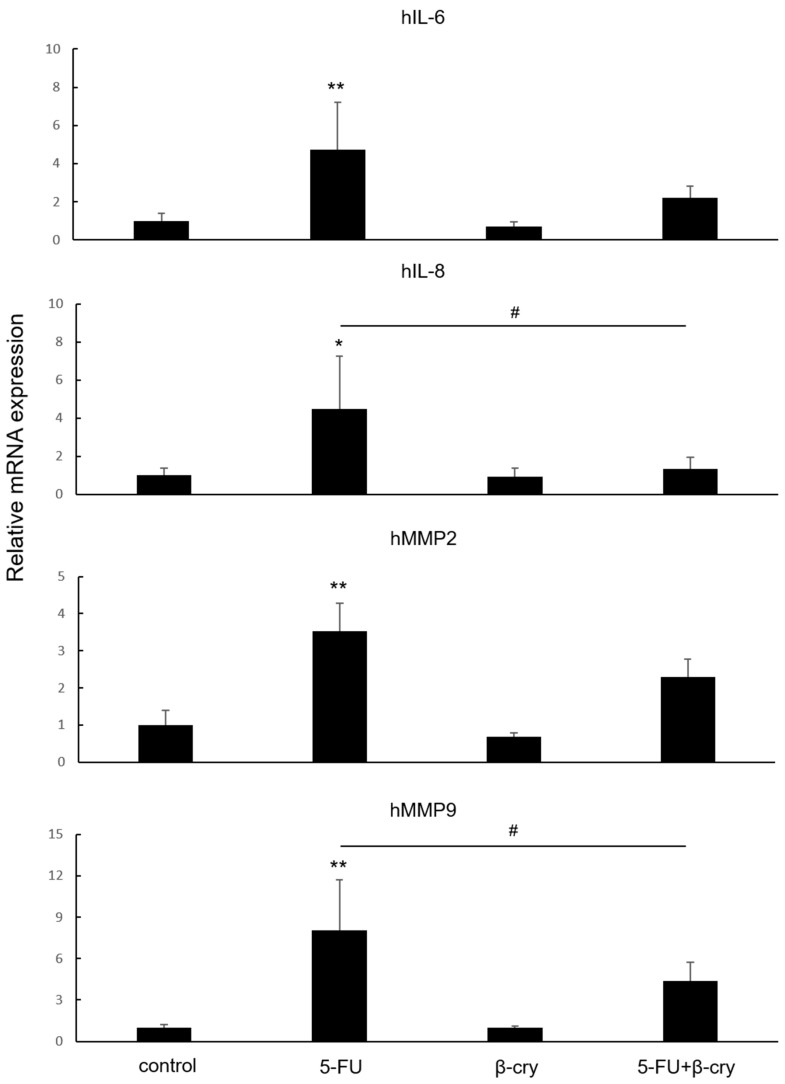
The effects of 5-FU and β-cry on the mRNA expression of inflammatory cytokines and MMPs in hOMK. The mRNA expression levels of IL-6, IL-8, MMP-2, and MMP-9 in 5-FU-treated hOMK were significantly higher than those in the control group (*p* < 0.05). The mRNA expression levels of IL-8 and MMP-9 in hOMK treated with 5-FU and β-cry were significantly lower than the 5-FU-treated hOMK (*p* < 0.05). * *p* < 0.05, ** *p* < 0.01 vs. control # *p* < 0.05 between each group.

The 5-FU group showed a significant increase in the mRNA expression levels of interleukin (IL)-6 (4.74 ± 2.47-fold), IL-8 (4.47 ± 2.76-fold), MMP-2 (3.52 ± 0.77-fold), and MMP-9 (8.07 ± 3.65-fold) compared with the control group. In contrast, the β-cry and 5-FU+β-cry groups showed no significant increases compared with the control group (Figure 2). The expression of IL-8 and MMP-2 mRNA was significantly decreased in the 5-FU+β-cry group (IL-8: 1.33 ± 0.60-fold, MMP-9: 4.35 ± 1.36-fold) compared with that in the 5-FU group (Figure 2).

### 2.3. Effects of β-Cry on 5-FU-Induced IL-6, IL-8, and ROS Production in hOMK

**Figure 3:**
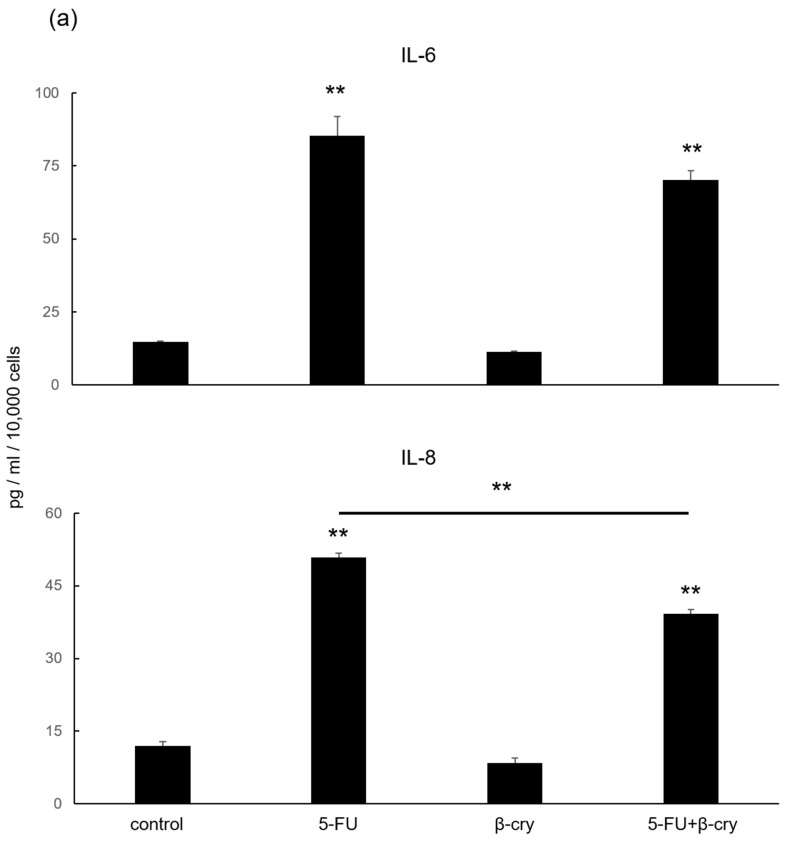

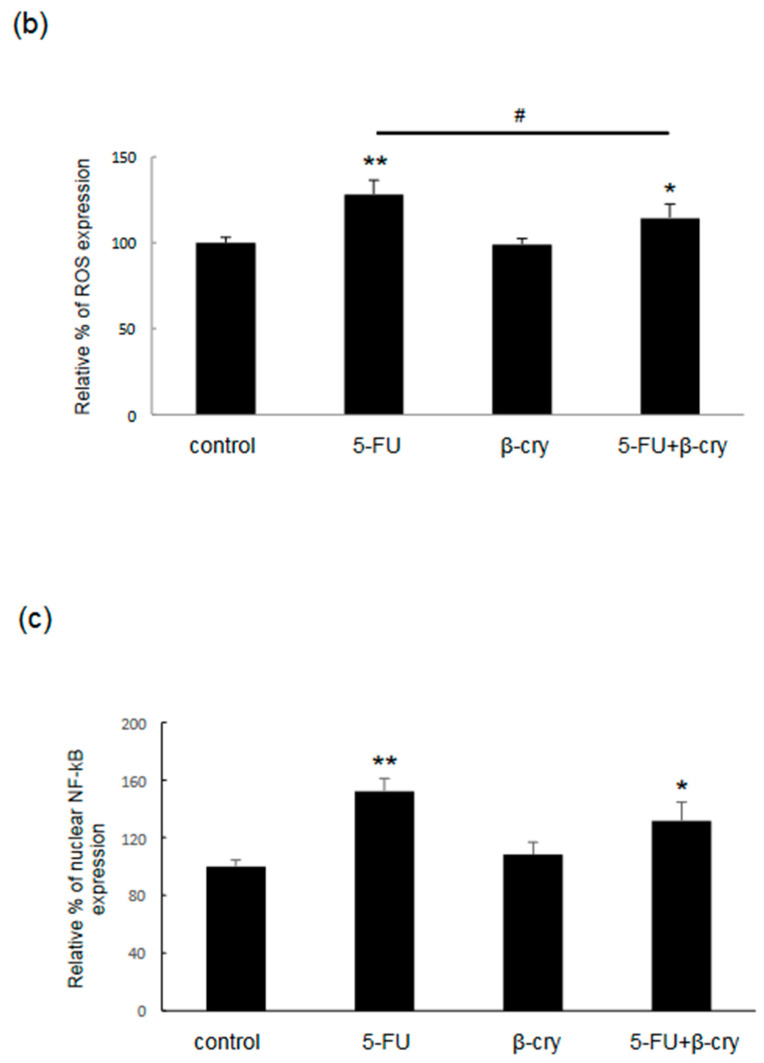
The effects of 5-FU and β-cry on the production of inflammatory cytokines and ROS in hOMK. (**a**) The protein production of IL-6 and IL-8 in 5-FU-treated hOMK and hOMK treated with 5-FU and β-cry was significantly higher than that in the control group (*p* < 0.01). The protein production of IL-8 in hOMK treated with 5-FU and β-cry was significantly lower than that in hOMK treated with 5-FU (*p* < 0.05). * *p* < 0.05, ** *p* < 0.01 vs. control # *p* < 0.05 between each group (**b**) The production of ROS in hOMK treated with 5-FU and hOMK treated with 5-FU and β-cry was significantly higher than that in the controls (*p* < 0.01). The production of ROS in hOMK treated with 5-FU and β-cry was significantly lower than that in hOMK treated with 5-FU (*p* < 0.05). * *p* < 0.05, ** *p* < 0.01 vs. control, # *p* < 0.05 between each group (**c**) Nuclear NF-κB expression in hOMK was significantly augmented by 5-FU treatment (*p* < 0.01). * *p* < 0.05, ** *p* < 0.01 vs. control.

A significant increase in IL-6 and IL-8 production (5-FU; IL-6: 85.31 ± 6.58 pg/mL, IL-8: 50.79 ± 2.90 pg/mL, 5-FU+β-cry; IL-6: 70.27 ± 3.01 pg/mL, IL-8: 39.14 ± 2.78 pg/mL) was observed in the 5-FU and 5-FU+β-cry groups compared with that in the control group (IL-6: 14.69 ± 0.35 pg/mL, IL-8: 11.88 ± 1.48 pg/mL). In contrast, no significant change was observed in the β-cry group (Figure 3a). In addition, IL-8 production was significantly reduced in the 5-FU+β-cry group compared with that in the 5-FU group (Figure 3a). Moreover, IL-6 and IL-8 production were significantly reduced in the 5-FU+Indometcin group compared with that in the 5-FU group, but no significant suppression was observed in the 5-FU+RA group (Supplementary Materials Figure S4). The 5-FU and 5-FU+β-cry groups showed a significant increase (127 ± 8.29%) in ROS production compared with the control group, while the 5-FU+βcry group showed a significant decrease (114.33 ± 8.16%) compared with the 5-FU group (Figure 3b).

### 2.4. Effects of β-Cry on 5-FU-Induced NF-κB Activation in hOMK

The 5-FU and 5-FU+β-cry groups showed a significant increase in the nuclear NF-κB expression (5-FU: 152.10 ± 9.00%, 5-FU+β-cry: 131.67 ± 12.80%) compared to the control group, while the β-cry group showed no significant change (Figure 3c). Although NF-κB expression was slightly decreased in the 5-FU+β-cry group compared with that in the 5-FU group, no significant difference was observed.

### 2.5. Effects of P. gingivalis Lipopolysaccharide (LPS) on IL-6 and IL-8 Production in 5-FU-Treated hOMK

**Figure 4:**
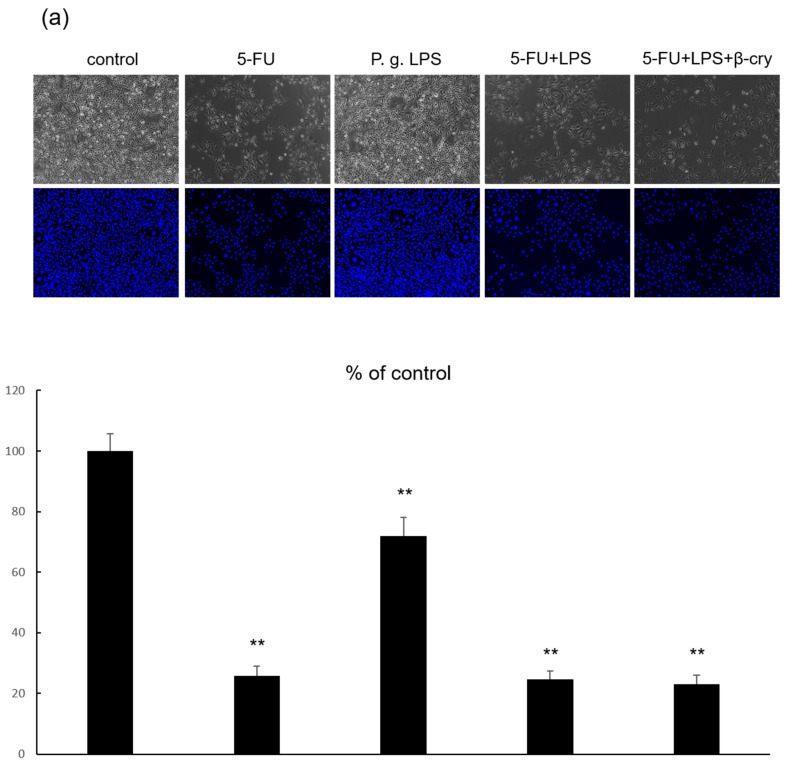

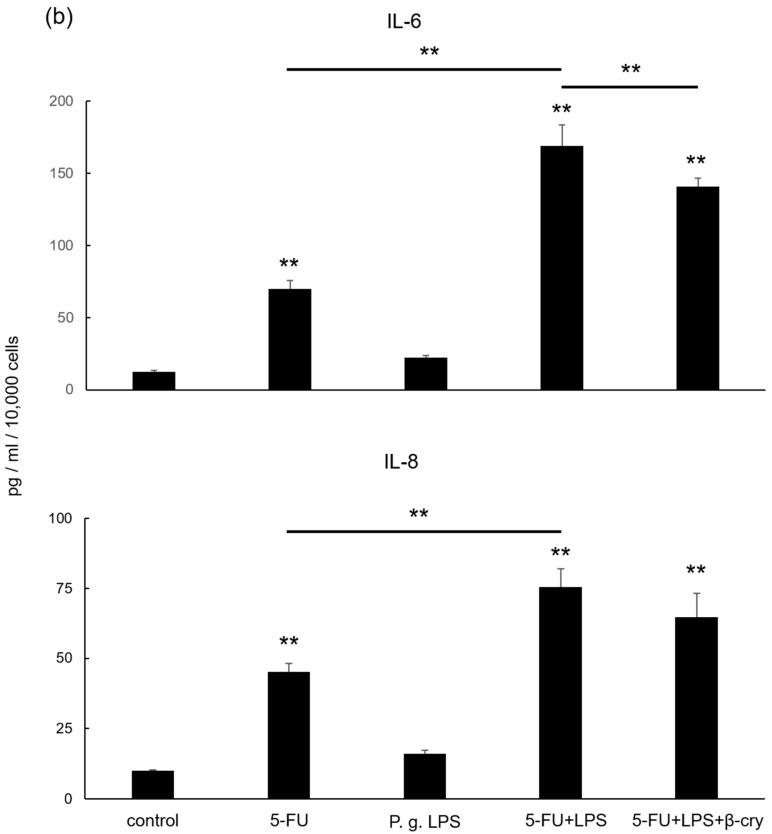
The effects of *P. gingivalis* LPS on the cell number and production of inflammatory cytokines in 5-FU-treated hOMK. (**a**) The cell number of hOMK treated with *P. gingivalis* LPS was significantly lower than that of the control group (*p* < 0.01). ** *p* < 0.01 vs. control (**b**) The protein production of IL-6 and -8 in hOMK treated with 5FU and *P. gingivalis* LPS were significantly higher than that in 5-FU-treated hOMK (*p* < 0.01). The protein production of IL-8 in hOMK treated with 5-FU, *P. gingivalis* LPS, and β-cry was significantly lower than that in hOMK treated with 5-FU and *P. gingivalis* LPS (*p* < 0.05). ** *p* < 0.01.

When hOMK were stimulated with 1 μg/mL of *P. gingivalis* LPS for 7 days, significant inhibition of cell proliferation (71.94% ± 6.20) was observed compared with that in the control group (Figure 4a). The 5-FU + *P. gingivalis* LPS group also showed a significant decrease in cell proliferation (24.53% ± 2.76) compared with the control group; however, the 5-FU group showed no significant difference (25.78% ± 3.87) (Figure 4a). In the *P. gingivalis* LPS group, IL-6 and IL-8 production were slightly increased (IL-6: 22.37 ± 1.35 pg/mL, IL-8: 15.96 ± 1.24 pg/mL), but the difference was not statistically significant compared with the control group (IL-6: 12.61 ± 0.87 pg/mL, IL-8: 10.03 ± 0.22 pg/mL) (Figure 4b). IL-6 and IL-8 production were significantly increased (IL-6 169.13 ± 14.57 pg/mL, IL-8 75.51 ± 6.64 pg/mL) in the 5-FU + *P. gingivalis* LPS group compared with that in the 5-FU alone group (IL-6: 70.05 ± 0.87 pg/mL, IL-8: 45.20 ± 3.00 pg/mL) (Figure 4b). The 5-FU + *P. gingivalis* LPS + β-cry group showed a significant decrease (140.78 ± 6.08 pg/mL) in IL-6 production compared with the 5-FU + *P. gingivalis* LPS group (Figure 4b).

## 3. Discussion

This study revealed that 5-FU strongly inhibited hOMK proliferation, provoked nuclear NF-κB activation, and up-regulated the production of MMPs, inflammatory cytokines, and ROS. Although β-cry enhanced the proliferation, it could not attenuate 5-FU-induced cell damage. However, β-cry down-regulated the expression of IL-8 and MMP-9 and IL-8 and ROS production induced by 5-FU treatment in oral mucosa-derived keratinocytes. In addition, *P. gingivalis* LPS treatment augmented 5-FU-induced IL-6 and IL-8 production.

The cytotoxic effects of 5-FU are produced by different metabolites produced intracellularly through the conversion of 5-FU to fluorodeoxyuridine monophosphate (FdUMP), fluorouridine triphosphate (FUTP), and fluorodeoxyuridine triphosphate (FdUTP) in the cell [28]. The thymidylate synthetase inhibitor 5-FU is widely used in the treatment of various cancers, including oral cancer, as it interrupts DNA synthesis, leading to cell death by apoptosis [29,30]. However, 5-FU often causes oral mucositis, leading to severe inflammation and ulceration in the oral mucosa as an adverse effect. NF-κB signaling reportedly plays a vital role in the pathobiology of mucositis, particularly via the up-regulation and subsequent expression of pro-inflammatory cytokines and MMPs [31,32]. The nuclear factor-κB (NF-κB) is a major transcriptional regulator of inflammatory cytokines (tumor necrosis factor-α/TNF-α, interleukin-6/IL-6, and interleukin-1β/IL-1β), cell adhesion molecules, stress response substances, and a major transcriptional regulator of >200 genes related to cytokine modulators [33,34]. Their expression can initiate epithelial signaling, thereby causing further tissue damage. Moreover, it has been suggested that pro-inflammatory cytokines were responsible for tissue damage in a patient with mucositis through a positive feedback loop mechanism [35]. Anticancer therapy also damages fibroblasts, which activates protein-1 (AP1), and secretes metalloproteinases (MMPs), such as MMP1 and MMP3, which degrade the collagenous subepithelial matrix and destroy the epithelial basement membrane, respectively [36,37]. As a result of the up-regulation of these pro-inflammatory cytokines and MMPs, widespread apoptosis occurs within the various epithelial and connective tissues. Thus, the expression of pro-inflammatory cytokines and MMPs plays an important role in the onset of mucositis. Previous studies examining the effects of 5-FU on oral-derived cells have reported that the stimulation of gingival fibroblasts with 5-FU causes a significant decrease in cell viability and a significant increase in ROS [38]. Moreover, the stimulation of oral cancer cells with 5-FU increases the production of IL-6 and IL-8 [39]. In addition, 5-FU administration reportedly causes tissue damage due to the increased expression of MMP-2 and MMP-9 [40]. These reports are consistent with the results of the present study. It has also been suggested that the oral flora is involved in the severity of chemotherapy-induced oral mucositis [41,42] and that *P. gingivalis*, in particular, inhibits wound healing and causes severe and prolonged chemotherapy-induced oral mucositis [43,44]. In the present study, the addition of *P. gingivalis* LPS with 5-FU increased the production of IL-6 and IL-8 from oral mucosal keratinocytes compared with stimulation with 5-FU alone, highlighting the importance of regular oral care in the prevention of chemotherapy-induced oral mucositis.

Recent reports suggest that adding an elemental diet to immortalized keratinocytes can inhibit nuclear NF-κB expression and inflammatory cytokine production induced by 5-FU [45], and the addition of vitamin E can inhibit ROS production induced by 5-FU [46]. Most in vitro, animal models, and human studies suggest that β-cry is better absorbed from its major food sources than any other common carotenoids [47,48,49]. Moreover, β-cry reportedly has anti-inflammatory [20,26,50] and antioxidant [51] effects, and its mechanism of action is through the inhibition of NF-κB activity via β-cry competing with the ATP-binding pocket and the attenuation of NFκB activation [20]. Furthermore, the p-p38 MAPK levels are reduced, and MAPKs are reportedly involved in the regulation of the NF-κB signaling pathway [52]. Regarding structure–activity relationships, β-cry is an important xanthophyll containing hydroxy and keto groups in its structure, one of few with pro-vitamin A activity, because it cleaves molecules in the middle to form retinal [53,54]. The central cleavage reaction, catalyzed by the cytoplasmic enzyme β-carotene 15,15′-oxygenase, oxidizes the C15-C15′ central carbon–carbon double bond in provitamin A carotenoids to produce retinal [55]. Cleavage of the C9′–C10′ double bond in various carotenoids, including non-provitamin A carotenoids, is asymmetrically catalyzed by β-carotene 9′,10′-oxygenase, another cleaving enzyme located in mitochondria [56,57]. B-cry showed a significant increase in mRNA expression of HoxA1, which is one of the vitamin A/retinoic acid-responsive genes, as well as retinoic acid (RA) (Supplementary Materials Figure S2). The conjugated double bonds in the structure enable carotenoids to accept electrons from reactive species, neutralize free radicals, and isomerize and protect autoxidation in the presence of oxygen, light, and heat [58,59]. Therefore, its structure protects against oxidative damage that can cause inflammation [60]. In addition, the chemical reactivity, distinctive shape, and light-absorbing properties of carotenoids are attributed to the alternating double and single bonds present in the nucleus of a polyene chain that constitute a conjugated system with delocalized π-electrons [61]. Carotenoids take up thermal energy from singlet oxygen and release this energy by polyene vibration [53]. They mimic enzymes, in that a single molecule of carotenoid can destroy hundreds of molecules of singlet oxygen [62]. Our results showed that β-cry increased cell proliferation and inhibited the 5-FU-induced expression of inflammatory cytokines, MMPs, ROS, and NF-κB activity similar to what has been previously reported [60,63,64,65].

The clinical symptoms of oral mucositis, such as ulceration of the mucosal epithelium, pain, infection, and swallowing dysfunction, are the results of an epithelial injury. Moreover, accumulating evidence indicates that the clinical manifestations of this condition are attributable to a series of interactive biological events that involve all cells and tissues of the mucous membrane. However, this was an in vitro study using only keratinocytes derived from the oral mucosa. Therefore, there are limitations in applying the results of this study directly to actual cases of oral mucositis. Further studies using oral mucosal keratinocytes and fibroblasts in a three-dimensional culture system and in vivo studies using experimental animal models of oral mucositis are required. In the future, vitamin supplementation in the form of tablets, capsules, and pastes should be used to evaluate success in both chemotherapy- and radiotherapy-induced oral mucositis [66,67,68,69]; hence, clinical trials in the form of long-lasting tablets are expected.

The results of the present study revealed that β-cry can enhance cell proliferation and down-regulate the 5-FU-induced expression of inflammatory cytokines and MMP in hOMK. Moreover, the inflammation induced by 5-FU in the oral mucosa was exacerbated by *P. gingivalis* LPS. These results suggest that β-cry application may help attenuate chemotherapy-induced oral mucositis, while concurrent oral hygiene treatment may be effective in managing oral mucositis.

## 4. Materials and Methods

### 4.1. Cell Culture

hOMK were purchased from Cell Research Corp. (Singapore, Republic of Singapore) and cultured in an epithelial culture medium (EpiLife basal medium supplemented with EpiLife Defined Growth Supplement, Thermo Fisher Science, Waltham, MA, USA), at 37 °C in a humidified atmosphere of 95% air and 5% CO_2_. hOMK were seeded at a density of 1, 3 × 10^4^, 1 × 10^5^, or 1 × 10^6^ cells/well in 96-well plates, 24-well plates, or six-well plates, depending on the experiment, and then stimulated with 5-FU ((0.5 μg/mL or 0.01–10 μg/mL) (Kyowa Kirin, Tokyo, Japan)) and β-cry ((1 × 10^−7^ M) (CaroteNature, Lupsingen, Switzerland)). In some experiments, the combination of LPS ((1 μg/mL) from *P. gingivalis* (InvivoGen, San Diego, CA, USA)), RA ((10 nM) (Sigma, St. Louis, MI, USA)) and indomethacin ((10 μg/mL) (Nacalai, Kyoto, Japan)) were also used and dissolved in DMSO.

### 4.2. Cell Viability Assay

hOMK were seeded in 96-well plates at a density of 1 × 10^4^ cells/well and stimulated with 5-FU (0.01–10 μg/mL) 24 h later. The cell activity was examined by WST assay (Cell Count Reagent SF, Nacalai Tesque, Kyoto, Japan) after 48 h. The absorbance was measured at 450–650 nm using a microplate reader (Emax^®^, Molecular Devices, Sunnyvale, CA, USA). The cell activity of each group is expressed as a percentage of the mean value of the no-stimulation group (control).

### 4.3. Cellular Growth and Morphology

hOMK were seeded in 24-well plates at a density of 3 × 10^4^ cells/well and stimulated with 5-FU (0.5 μg/mL) and β-cry (1 × 10^−7^ M) 24 h later. Seven days later, the cells were fixed in 10% formalin (Nacalai Tesque, Kyoto, Japan), nuclear stained with Hoechst 33342 (Dojindo, Kumamoto, Japan), and the cell morphology was observed using an inverted fluorescence phase contrast microscope (BZ-X810, Keyence, Japan) and cell counts were taken. The average number of cells in the control was set as 100%, and the number of cells in each group was expressed as a percentage. For cell morphology, the long and short axes were measured and evaluated in terms of the ratio of the major to minor axes.

### 4.4. Quantitative Real-Time Reverse Transcriptase Polymerase Chain Reaction (RT-PCR)

hOMK were seeded in 24-well plates at a density of 3 × 10^4^ cells/well and stimulated with 5-FU (0.5 μg/mL), β-cry (1 × 10^−7^ M), and *P. gingivalis* LPS (1 μg/mL) 24 h later. RNA was collected from the cells after 7 days and isolated, and cDNA was synthesized according to a method previously described [40,41]. Briefly, the total RNA was extracted using RNeasy mini kit (Qiagen, Venlo, The Netherlands) and reverse-transcribed using ReverTra Ace qPCR RT Master Mix (Toyobo, Osaka, Japan). RT-PCR was performed using Real-Time PCR Master Mix (Applied Biosystems, Waltham, MA, USA) and matching probes and primers (Thermo Fisher Science) on a Step One Plus Real-Time PCR System (Applied Biosystems). All values (average ± standard deviation (SD)) were normalized with respect to the β-actin mRNA level in each sample and expressed as relative values.

### 4.5. Enzyme-Linked Immunosorbent Assay (ELISA)

hOMK were seeded in 24-well plates at a density of 3 × 10^4^ cells/well and stimulated with 5-FU (0.5 μg/mL), β-cry (1 × 10^−7^ M), and *P. gingivalis* LPS (1 μg/mL) 24 h later. After 7 days, culture supernatants were collected, and the production of cytokines (IL-6 and IL-8) in the culture supernatants was measured using an ELISA kit (R&D Systems, Minneapolis, MN, USA). The absorbance was measured at 450–650 nm using a microplate reader (Emax^®^, Molecular Devices, Sunnyvale, CA, USA), and the concentration of each sample was calculated from the standard curve. The value from each sample is shown per 10,000 cells.

### 4.6. ROS Assay

ROS assays (Cell MeterTM Fluorimetric Intracellular Total ROS Activity Assay Kit, AAT Bioquest, Sunnyvale, CA, USA) were conducted in accordance with the manufacturer’s instructions.

hOMK were seeded in 24-well plates at a density of 1 × 10^5^ cells/well and stimulated with 5-FU (0.5 μg/mL) and β-cry (1 × 10^−7^ M) in the presence of Amplite™ ROS green working solution for 8 h after 24 h. The fluorescence values of each sample were measured (Excitation: 490 nm, Emission: 525 nm, Cutoff: 515 nm) with a SpectraMax M2, Molecular Devices, Tokyo, Japan). The fluorescence intensity of each group was expressed as a percentage of the mean value of the no-stimulation group (control).

### 4.7. NF-κB Activity Assay

The NF-κB activity was measured using the TransAM NF-κB p65 kit, according to the manufacturer’s instructions (Active Motif, Carlsbad, CA, USA).

hOMK were seeded in six-well plates at a density of 1 × 10^6^ cells/well and stimulated with 5-FU (0.5 μg/mL) and β-cry (1 × 10^−7^ M) for 24 h. After 1 h, the cells were stripped using a cell scraper and treated with Nonident P-40 to remove the intracytoplasmic components. The nuclear components of the sample cells were later collected in the lysis buffer provided. The samples were analyzed in a 96-well plate containing the immobilized NF-κB consensus site (5′-GGGACTTTCC-3′) oligonucleotide. The active forms of NF-κB p-65 in the nuclear extracts were bound to the oligonucleotides on the plate and detected calorimetrically by a microplate reader (Emax^®^, Molecular Devices, Sunnyvale, CA, USA) at an absorbance of 450 nm and with a reference wavelength of 650 nm.

NF-κB activity is expressed as 100% for the no-stimulation group (control), and NF-κB activity in each group is expressed as a percentage of the NF-κB activity of the control group.

### 4.8. Statistical Analysis

Data are expressed as mean ± SD. Statistical significance was analyzed using Student’s *t*-test and analysis of variance with Tukey–Kramer post hoc test. *p* < 0.05 was considered significant.

## 5. Conclusions

This study demonstrates that β-cry promotes cell proliferation in hOMK and that β-cry can suppress the expression of inflammatory cytokines and MMPs induced by 5-FU. Furthermore, the inflammation induced by 5-FU in the oral mucosa was exacerbated by *P. gingivalis* LPS. Taken together, these findings suggest the possibility that β-cry can alleviate the symptoms of chemotherapy-induced oral mucositis and that its combination with oral care is effective in the management of oral mucositis.

## Data Availability

Not applicable.

## Supplementary Materials

The following supporting information can be downloaded at: https://www.mdpi.com/article/10.3390/molecules28072935/s1, Figure S1: Cellular activity of hOMK; Figure S2: Homeotic gene cluster (Hox) genes as vitamin A/retinoic acid-responsive genes expression; Figure S3: Cellular morphological changes in hOMK; Figure S4: Effects of indomethacin used as a standard drug for oral mucositis; Figure S5: Structural formula of β-cryptoxanthin (β-cry).

## Sample Availability

Samples of the compounds are not available from the authors.
