# Supplementary material for: Anti-Inflammatory Effects of β-Cryptoxanthin on 5-Fluorouracil-Induced Cytokine Expression in Human Oral Mucosal Keratinocytes
Source: Molecules. 2023 Mar 24;28(7):2935. doi: 10.3390/molecules28072935 (PMC10095812; doi:10.3390/molecules28072935)
Supplement: Supplementary file 1 [file molecules-28-02935-s001.zip › molecules-2071918-supplementary.pdf]

molecules

MDPI

Supplementary Materials

Anti-Inflammatory Effects of β-Cryptoxanthin on 5-Fluorouracil-Induced Cytokine Expression in Human Oral Mucosal Keratinocytes

Hironaka Yamanobe [1,2], Kenta Yamamoto [1,2,*], Saki Kishimoto [1], Kei Nakai [1], Fumishige Oseko [1], Toshiro Yamamoto [1], Osam Mazda [2] and Narisato Kanamura [1]

1 Department of Dental Medicine, Kyoto Prefectural University of Medicine, Kyoto, 602-8566, Japan;
2 Department of Immunology, Kyoto Prefectural University of Medicine, Kyoto, 602-8566, Japan
* Correspondence: fiori30@koto.kpu-m.ac.jp; Tel.: +81-75-251-5329

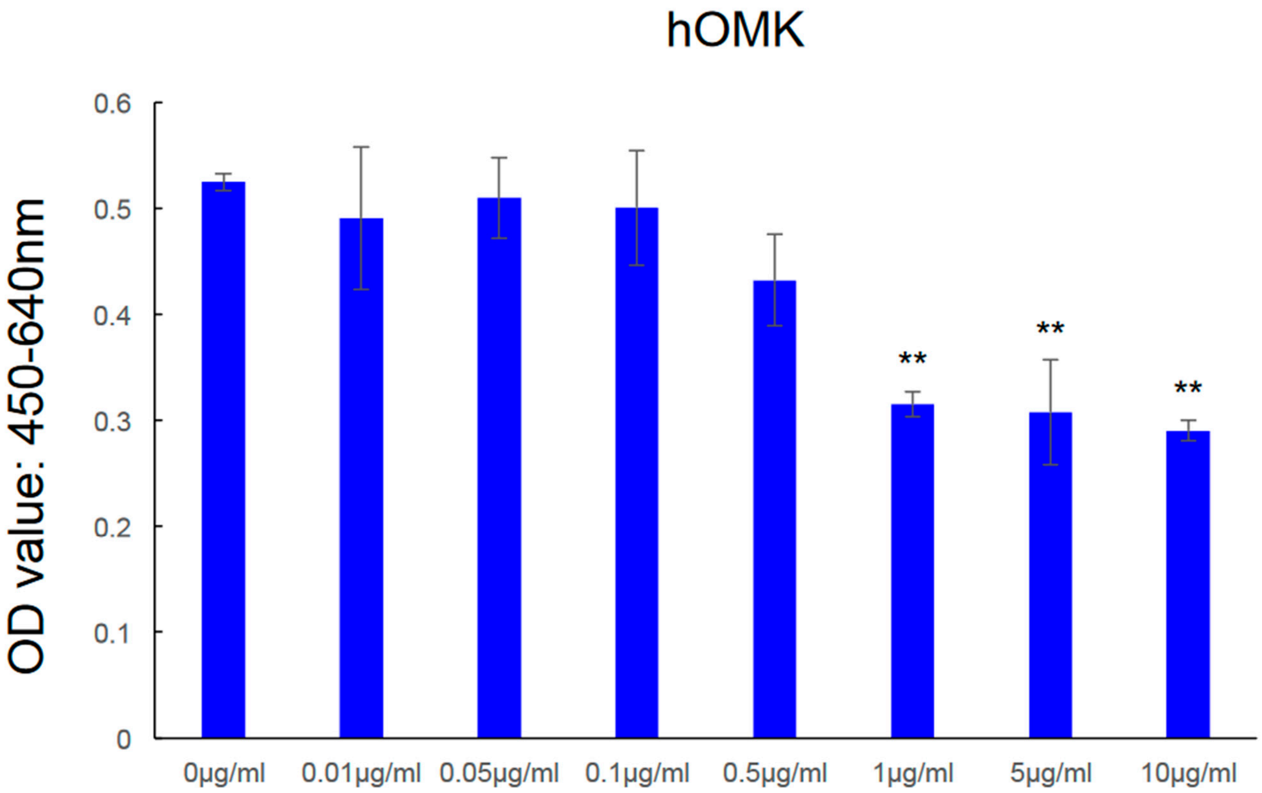

Figure S1. Cellular activity of hOMK 5-fluorouracil (5-FU) concentration above 1 μg/mL showed a significant decrease in cell growth ($p<0.01$).

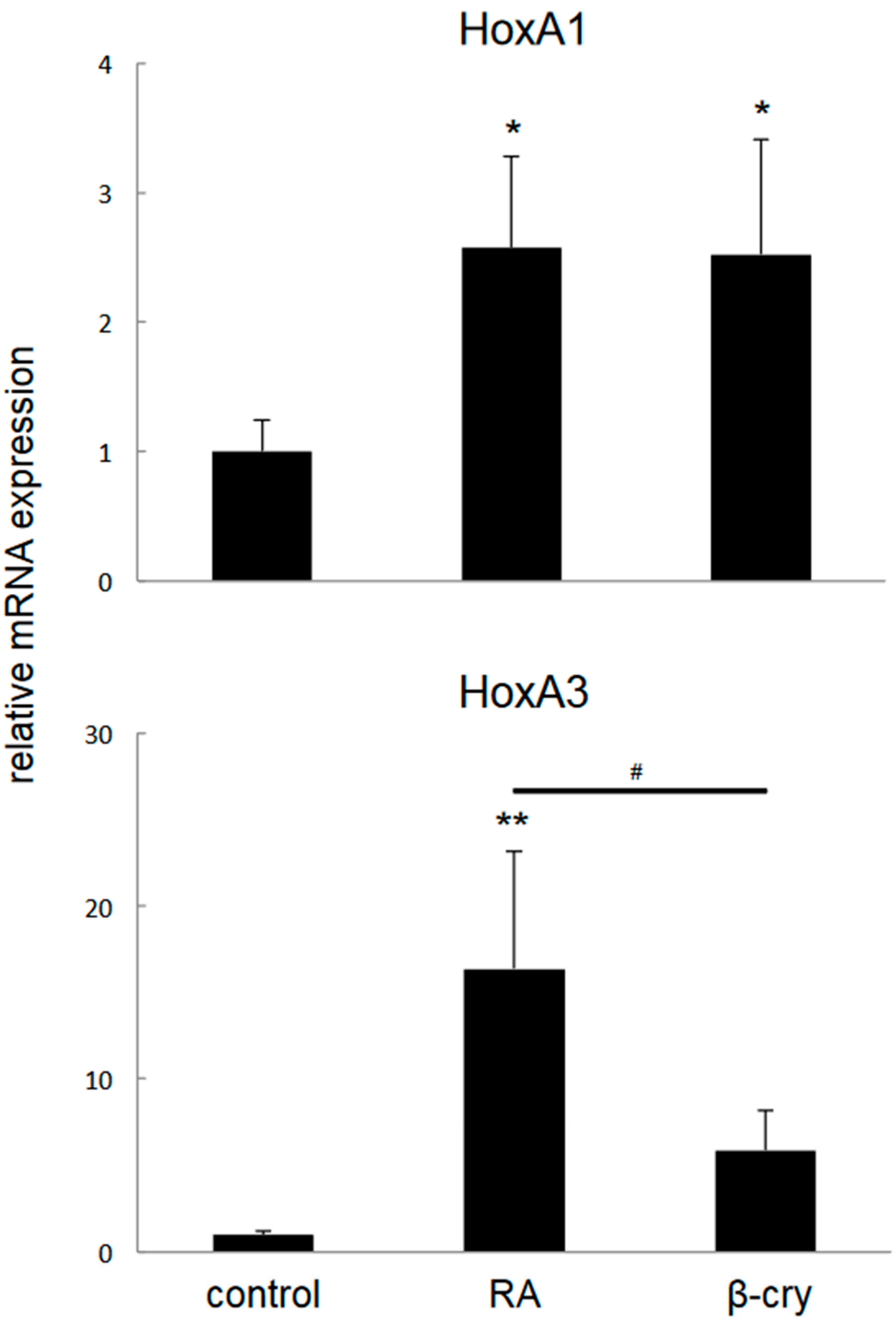

Figure S2. Homeotic gene cluster (Hox) genes as vitamin A/retinoic acid-responsive genes expression.β-cryptoxanthin (β-cry) showed a significant increase in mRNA expression in HoxA1 as well as retinoic acid (RA) ($p<0.05$).

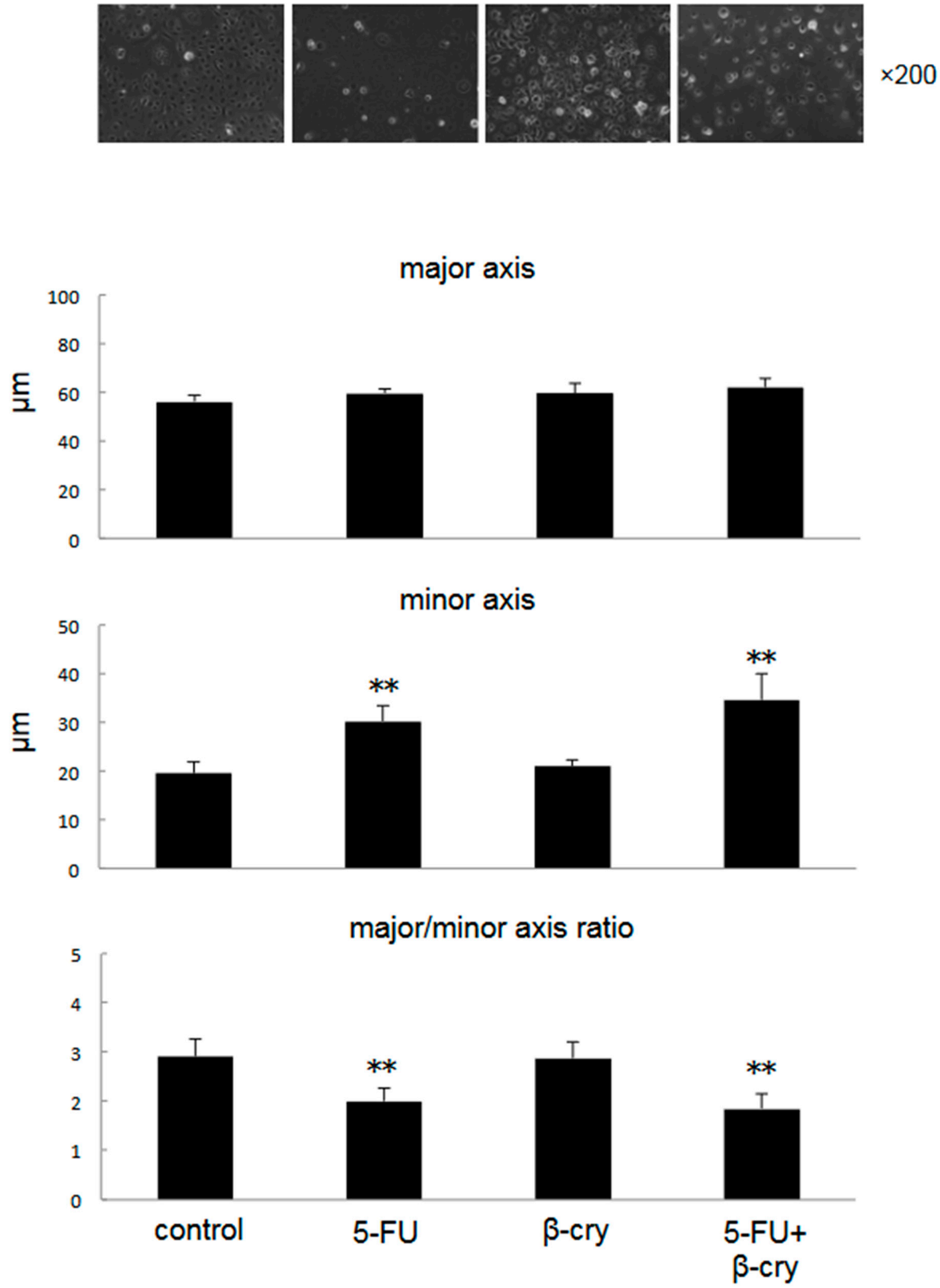

Figure S3. Cellular morphological changes in hOMK. The long-short axis ratio was significantly reduced in the 5FU-stimulated and 5FU+ β-cry-stimulated groups compared to the control group ($p<0.01$).

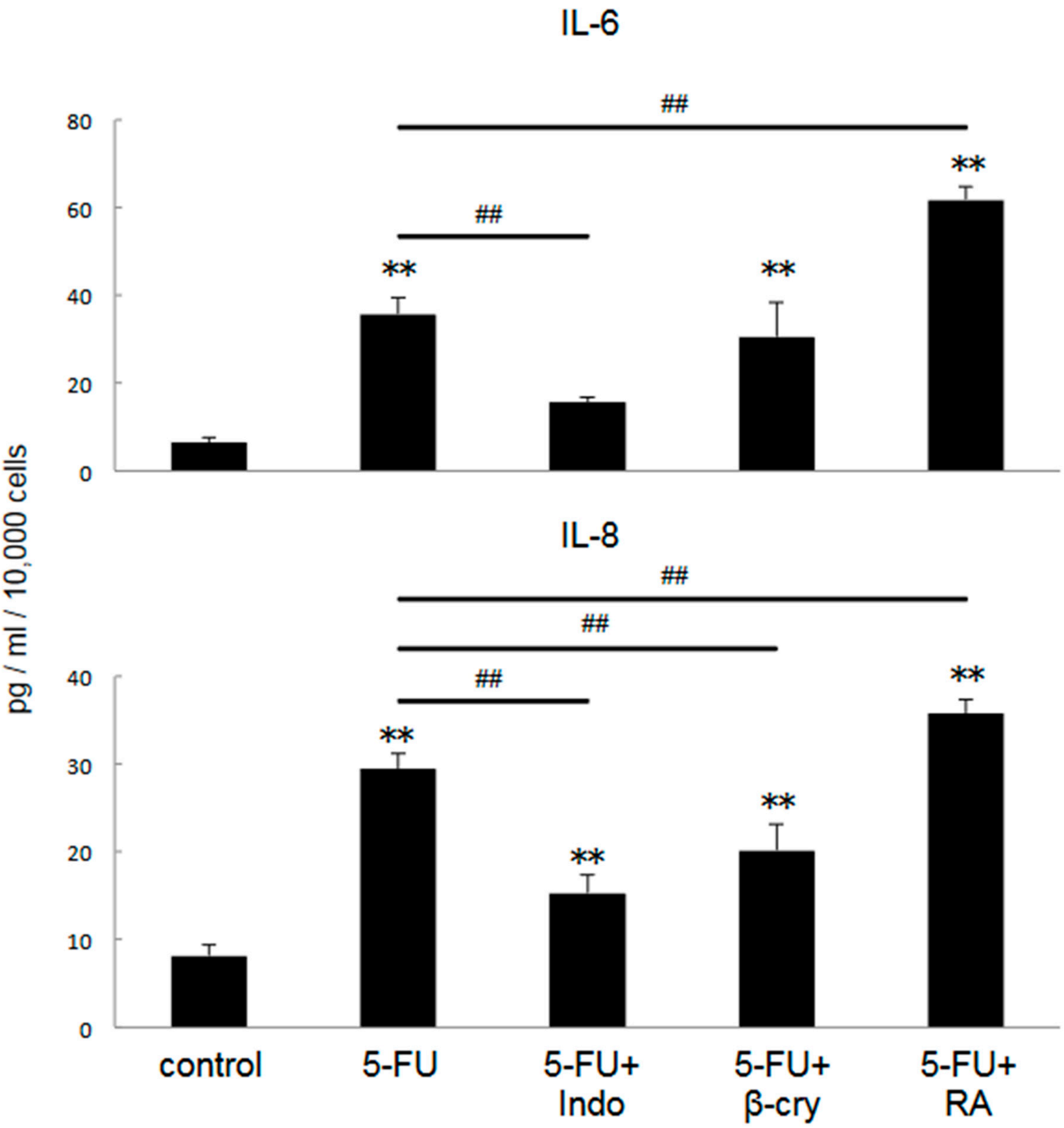

Figure S4. Effects of indomethacin used as a standard drug for oral mucositis. IL-6 and IL-8 production was significantly reduced in the 5-FU+Indometcin group compared with that in the 5-FU group (p<0.01). In addition, IL-8 production was significantly reduced in the 5-FU+β-cry group compared with that in the 5-FU group (p<0.01).

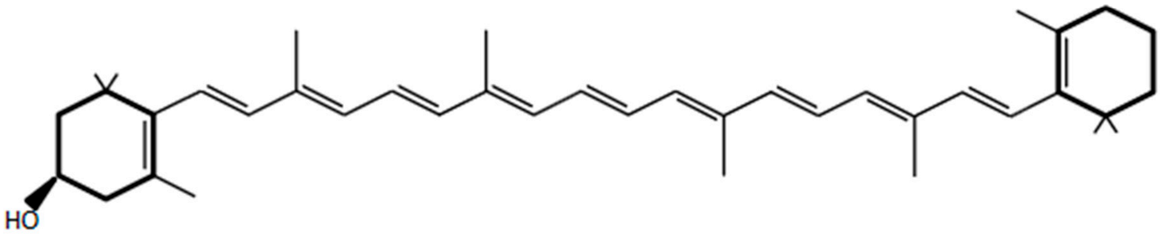

Figure S5. Structural formula of β-cryptoxanthin (β-cry).
